# Trends in Drug Delivery Systems for Natural Bioactive Molecules to Treat Health Disorders: The Importance of Nano-Liposomes

**DOI:** 10.3390/pharmaceutics14122808

**Published:** 2022-12-15

**Authors:** Raiane Vieira Cardoso, Patricia Ribeiro Pereira, Cyntia Silva Freitas, Vania Margaret Flosi Paschoalin

**Affiliations:** Programa de Pós-Graduação em Ciência de Alimentos e Programa de Pós-Graduação em Quimica, Instituto de Química, Universidade Federal do Rio de Janeiro, Av. Athos da Silveira Ramos 149-sala 545-Cidade Universitária, Rio de Janeiro 21941-909, RJ, Brazil

**Keywords:** nano-delivery systems, liposomal formulations, liposomal marketed formulations, liposome–cell interaction, protein and peptide encapsulation

## Abstract

Drug delivery systems are believed to increase pharmaceutical efficacy and the therapeutic index by protecting and stabilizing bioactive molecules, such as protein and peptides, against body fluids’ enzymes and/or unsuitable physicochemical conditions while preserving the surrounding healthy tissues from toxicity. Liposomes are biocompatible and biodegradable and do not cause immunogenicity following intravenous or topical administration. Still, their most important characteristic is the ability to load any drug or complex molecule uncommitted to its hydrophobic or hydrophilic character. Selecting lipid components, ratios and thermo-sensitivity is critical to achieve a suitable nano-liposomal formulation. Nano-liposomal surfaces can be tailored to interact successfully with target cells, avoiding undesirable associations with plasma proteins and enhancing their half-life in the bloodstream. Macropinocytosis-dynamin-independent, cell-membrane-cholesterol-dependent processes, clathrin, and caveolae-independent mechanisms are involved in liposome internalization and trafficking within target cells to deliver the loaded drugs to modulate cell function. A successful translation from animal studies to clinical trials is still an important challenge surrounding the approval of new nano-liposomal drugs that have been the focus of investigations. Precision medicine based on the design of functionalized nano-delivery systems bearing highly specific molecules to drive therapies is a promising strategy to treat degenerative diseases.

## 1. A General Overview of the Remarkable Role of Nature in Providing Potential Pharmacological Compounds

**Figure 1:**
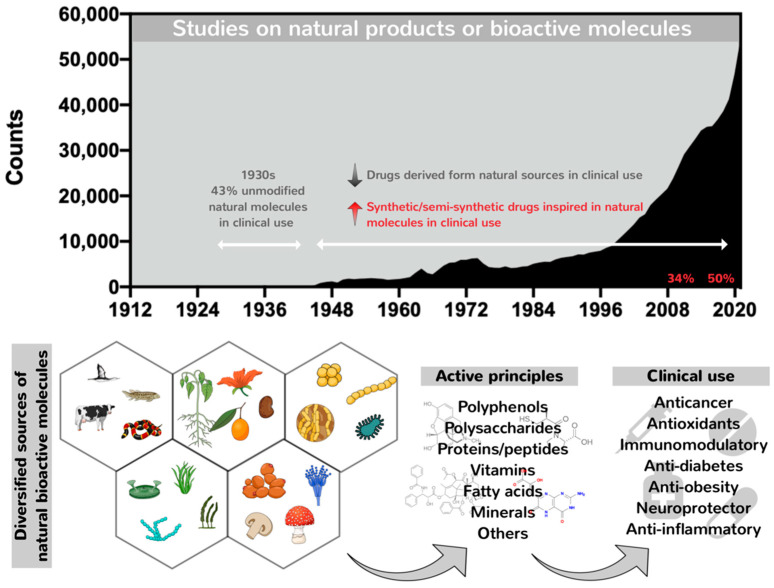
Number of reports on bioactive molecules and natural products over the years, retrieved by an advanced search on the Pubmed database (https://pubmed.ncbi.nlm.nih.gov accessed on 5 July 2022) combining the terms “natural products” OR “bioactive molecules”. The number of publications contrast with FDA-approved unmodified natural drugs, which reached 43% in the 1930s. With the emergence of modern high-throughput platforms for screening and synthetic combinatorial strategies, synthetic/semi-synthetic drugs inspired by natural molecules reported throughout the years predominate over unmodified natural drugs in the market, increasing from 34% in 2010 to 50% currently.

Nature has long proved an outstanding source of bioactive molecules with vast structural complexity and diversity. These have been used for millennia, mainly as component mixtures in crude extracts, as a unique strategy to treat several physiopathological conditions, including wounds, infectious diseases and other disorders [1,2]. In contrast, conventional drugs approved for therapeutic use are mainly represented by synthetic and small molecules ranging from 900 to 1500 Da designed and chemically synthetized to fit a pre-determined target via high-throughput screening. However, since the targets for small therapeutic molecules represent only 2–5% of the human genome products, the search for alternative therapeutics capable of expanding the number of new targets has increased the number of studies on natural therapeutic biomolecules, comprising searches in diverse classes, including natural macromolecules particularly proteins, peptides and polysaccharides [3,4]. The first efforts to identify and isolate active principles from natural extracts began in the 19th century with the isolation of the anti-malarial quinine, the opiate analgesic morphine and salicylic acid, creating the medicinal use of naturally isolated compounds. Since then, many other bioactive compounds have been isolated, mainly alkaloids, such as caffeine, nicotine, codeine, atropine, colchicine, cocaine and capsaicin [5]. The utilization of unmodified natural molecules increased by 43% during the 1930s (Figure 1). After this period, a decline in the number of unmodified natural products entering clinical practices took place, although the number of published data on natural products and bioactive molecules has increased. The past 30 years have been marked by a profile shift characterized by the replacement of unmodified natural molecules by semisynthetic and synthetic derivatives chemically designed but inspired by natural molecules (Figure 1) [6]. This profile shift was stimulated by the emergence of modern high-throughput platforms for screening and synthetic combinatorial strategies aimed at the fast discovery of new drug candidates similar to or analogs of natural molecules displaying special activities identified over the years, conducted through semi-synthetic modifications or by total synthesis, but still maintaining their pharmacophore. Moreover, the generation of synthetic/semisynthetic analogs is in contrast to time-consuming and high-cost methodologies applied to the identification of natural compounds in crude extracts, followed by their isolation and obtention in bulk amounts, impacting the emergence of new but unmodified natural pharmacological compounds for clinical use [7,8,9]. Because of this, the chemical synthesis of natural products was prioritized to optimize pharmaceutical production, improving the purity, quality and yields of bioactive compounds with reduced costs. Salicylic acid was the first natural product synthesized in 1853, copying the natural molecule [5].

The number of compounds approved by the FDA for clinical use classified as natural-based synthetic and semi-synthetic and purely natural compounds comprised 34% of the top 100 best-selling medicines in 2010, increasing to about 50% today, as a result of the cumulative knowledge reported in studies on bioactive molecules over the past 20 years, expanding the hall of new pharmaceutical molecules (Figure 1) [3,10].

Natural bioactive compounds can be found in living organisms from both edible or non-edible sources, mainly plants, including fruits, vegetables, whole grains, legumes, oils and others, but also animals and microorganisms such as bacteria and fungi, as well as algae [11]. The most studied bioactive compounds are polyphenols, such as curcumin, resveratrol, epigallocatechin gallate (EGCG), quercetin and anthocyanin, followed by polyunsaturated fatty acids such as docosahexaenoic acid (DHA), eicosapentaenoic acid (EPA) and arachidonic acid (ARA). However, native proteins or enzymes, as well as their bioactive peptides encrypted in primary structures, are being considered the next generation of pharmacological compounds. Several studies have been developed addressing plant lectins and milk proteins as non-toxic but highly effective drugs to treat several diseases. Moreover, bioactive polysaccharides, including lentinan and grifolan, vitamins, carotenoids, alkaloids and even mineral elements such as Zn, Fe, Mg, Ca, Na and P can have a positive impact on human health. These natural compounds exhibit various health-promoting effects such as antioxidant [12], antitumoral [13], immunomodulatory [14], anti-diabetic and anti-obesity [15,16], antiviral [17], anti-inflammatory [18] and neuroprotector activity [19], among others.

Therefore, natural bioactive compounds and their structural analogs—the natural-inspired molecules—have profoundly impacted the history of drug discovery, especially concerning cancer and infectious diseases, and continue providing pharmaceuticals able to treat almost all health disorders while composing the hall of new active agents in their unaltered, synthetic or semi-synthetic forms [1,8,9].

The short half-life of natural bioactive compounds in the blood stream or human tissues is maybe the main obstacle toward their use as medicines due to their chemical instability toward processing and storage conditions, as well as easy degradation, which may be associated with impaired permeability and low bioavailability and absorption, compromising the target drug moiety [20]. Drug delivery systems have long emerged as an alternative to improve bioactive compound pharmacokinetics and therapeutic indices, leading to their desired performance.

## 2. Evolution of Drug Delivery Systems and the Emergence of Nanotechnology in Clinical Treatments

**Figure 2:**
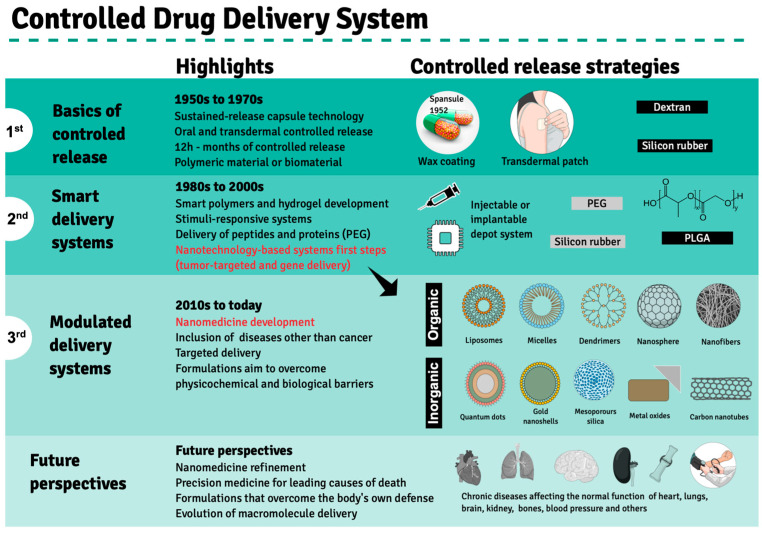
Development of controlled drug delivery systems. Three distinct development phases—1st, 2nd and 3rd generations—are highlighted based on therapeutic products approved by the FDA since the 1950s, when Spansule^®^, the first drug release system, was approved for clinical use. The main highlights of each phase are depicted in the central column, while the main synthetic or biological material used in the drug delivery system preparation is indicated in the left column. The advent of nanotechnological drug delivery strategies is highlighted in red during the last decade of the second development phase. Future perspectives are exhibited at the bottom panel. Created using Mind the Graph (https://mindthegraph.com accessed on 14 August 2022).

Drug performance depends primarily on the type of delivery and release rate in order to guarantee a sustained drug amount that matches the therapeutic index between the maximum safe concentration and above the minimum effectiveness in the blood stream, avoiding abrupt peak concentrations as a result of massive drug release or the need for multiple doses to achieve drug effectiveness. These limitations encouraged the search for an ideal drug delivery system to substitute pills and capsules that release active compounds in aqueous media, termed immediate release (IR) formulations. The pioneer strategy in this regard comprised Spansules^®^, a first-generation drug delivery system, where micro-pellets were coated with a water-soluble wax of varied thicknesses, allowing for the controlled release of oral drugs for up to 12 h, leading to a constant concentration in blood. The first-generation drug delivery period, which ran from the 1950s to the 1970s, was also marked by the development of silicon- or dextran-based transdermal formulations based on controlled release, mainly through dissolution and diffusion (Figure 2). Both delivery systems faced several challenges, leading to advanced-generation systems [21].

The second generation of drug delivery formulations began in 1980 and lasted until 2010 and came with the understanding that it is not indispensable that drug concentrations be maintained at fixed levels. This period was marked by the development of smart polymers and hydrogels that evolved alongside biodegradable microparticles, solid implants and in situ gel-forming implants capable of delivering long-term release and stimuli-responsive bioactive compounds. Finally, nano-delivery systems emerged, where nanoparticles obtained from biodegradable polymers, polymeric micelles, lipids, chitosan and dendrimers are used to carry anticancer agents and gene sequences. The need to overcome biological and physicochemical barriers gave rise to the third generation of drug delivery systems, in which advanced nanomaterials enable the delivery of poorly water-soluble and/or very labile drugs or cell components, including peptides, proteins and DNA or RNA sequences. Novel delivery concepts include targeted drug delivery using nanoparticles and self-regulated drug delivery [21]. However, few drugs were approved by the FDA, even though much experimental evidence has proven that drug delivery employing nanotechnology systems could enhance the effectiveness of anticancer drugs against tumors in animal models [10].

According to the National Nanotechnology Initiative (2021), nanotechnology involves the manipulation of nanoparticles ranging from 1 to 100 nm, and their use is widespread in several areas, including engineering, physics and informatics. However, nanotechnology applications for pharmaceutical purposes may cause the most significant impact on human health and, because of this, has been considered the most promissory technology for treatments against degenerative pathologies such as cancer [22,23] and central nervous system disorders [24,25], as well as in antiviral therapy to aid in SARS-CoV-2 immunization and treatment [26,27].

The morphology of nano-scaled particles led to the optimization of treatments by enabling nano-materials to reach physiological sites that used to remain inaccessible, such as specific areas of the brain damaged by synucleinopathies or brain neoplasms, which require the ability to cross the blood–brain barrier to achieve therapeutic intervention success [25]. Moreover, nanomaterials exhibit a large surface/volume ratio, potentiating drug effects on target sites (cell, tissue or organ), comprising efficient bioactive molecule nanocarriers that can be encapsulated through adsorption, core entrapment or covalent surface binding. The nano-encapsulated bioactive molecule is released constantly and in a controlled manner, reducing adequate drug dosages to achieve pharmacological effects and minimize side effects widely attributed to conventional pharmaceuticals [28,29,30].

Nano-encapsulated compounds can be protected from degradation in the bloodstream. They can still reach intracellular compartments via endosomes through passive permeability, releasing bioactive compounds into the cytoplasm or directing them to intracellular targets with ligands associated with nano-capsule internalization [31]. In anti-tumorigenic therapies, the passive accumulation of anticancer drugs at the tumor localization can be increased by facilitating the permeability and retention effect (EPR), a phenomenon following the local inflammatory status of tumor blood vessels that become leaky, allowing for the passage and accumulation of nanometric materials in tumor tissues [32,33,34,35].

The primary chemical composition of nanoparticles described for medicinal therapy treatments comprises organic or inorganic compounds. The former includes natural or synthetic polymers such as chitosan, collagen, glycerol, polylactic-co-glycolic acid (PLGA) and dendrimers, as well as lipid-based materials, such as liposomes and micelles. At the same time, inorganic nanoparticles comprise gold nano-shells, metal oxides, carbon nanotubes and quantum dots (Figure 2) [36]. Molecules for therapeutic purposes can be encapsulated by these nanoparticles or complexed to them by adsorption or covalent binding to the nanocarrier surface. Depending on the carried bioactive compound, these nanoconjugates may be used to treat several pathologies, including degenerative diseases such as atherosclerosis and Parkinson’s disease, as well as cancers, as mentioned previously (Table 1).

The application of hydrophobically modified chitosan nanoparticles in the delivery of silibinin, a flavo-lignan isolated from the seeds of the milk thistle plant, was shown to improve the response promoted by a sustained release and enhance the solubility of this poorly aqueous soluble compound [47]. Similarly, resveratrol (RV), a polyphenol non-flavonoid commonly found in red or dark grapes, encapsulated in albumin nanoparticles (NPs) and functionalized with the tripeptide arginine-glycine-aspartate (RGD), demonstrated a prolonged RV blood circulation time and increased content surrounding a target tumor by 8.1-fold compared to free-RV. In a murine model, RV-NPs-RGD suppressed tumor growth with no relapse after 35 days of treatment, while progressive tumor growth was observed in an RV-free treatment, indicating that RV-NPs-RGD should be considered a promising chemotherapy agent [48,49].

Curcumin, another widely studied natural antioxidant and anticancer compound derived from turmeric or saffron, displays limited clinical application due to its molecule instability and poor solubility. However, curcumin encapsulated in solid lipid nanoparticles displays a high antiproliferative effect against SKBR3 cancer cells compared to free curcumin, with nanoencapsulation improving this compound’s bioavailability. Moreover, nano-encapsulated curcumin induced high-extension SKBR3 cell apoptosis (36.7%) and pronounced cell migration inhibition [50].

The co-encapsulation of tocotrienol (TRF) and simvastatin (SIM) in lipid nanoparticles has been reported as displaying an anti-proliferative effect on the breast adenocarcinoma +SA cell line, with an IC_50_ of 0.52 μM, when compared to each pharmaceutical separately. Nearly 25 to 40% of SIM is released over 48 h, with a quicker release in the first 10 h, followed by a slower and controlled kinetic release throughout the following 38 h [37]. Similarly, when lianol (LN) was encapsulated in solid nanoparticles, it affected HepG2 hepatocarcinoma and A549 pulmonary adenocarcinoma cell lines [38].

Folic acid conjugated through ionic interactions with chitosan nanoparticles (FA-CS) loaded with vincristine induces apoptosis in 75% of NCI-H460 lung cancer cells, in contrast to unloaded nanoparticles (31%) [40]. Nanoencapsulation efficiency can be increased when prepared at 4:25 (*v*/*v*) of vincristine/FA-CS, reaching a 95% efficiency and loading capacity of 48.65%, reinforcing promising anticancer effects on tumorigenic lung cells [40]. Similar effects were observed using PLGA (poly lactic-co-glycolic acid) nanofibers coated with metformin (MET), which exhibited cytotoxicity against A549 lung cancer cells after 48 h compared to free MET [43].

Liposome encapsulation improves the pharmacological effects of bioactive molecules, including metallic complexes, contributing to toxicity constraints by decreasing effective doses. Encapsulation of iridium III complexes (Ir-1, Ir-2 and Ir-3) in nano-liposomes, for example, was shown to improve their anticancer activity against several human carcinoma lineages, such as HepG2, hepatocellular carcinoma; HTC-116, colon cancer; HeLa, cervical cancer; A549, lung carcinoma; BEL-7402, hepatocellular carcinoma; SGC- 7901, gastric adenocarcinoma; and Eca-109, esophagus cancer cell, and against B16 mouse melanoma cells but no toxicity against healthy nih3T3 murine cells. On the other hand, no activity was observed for free Ir-2, and the nano-liposomal formulation exhibited a superior IC50 compared to cisplatin, especially against the A549 cell line. The intraperitoneal administration of the nano-liposomal formulation loaded with the Ir-2 complex for 10 consecutive days prior to A549 carcinogenic cell inoculation in mice resulted in 57.45% tumor mass reduction. In vitro assays have shown that encapsulated complexes stimulate apoptotic activity induced by increased intracellular ROS and cell cycle arrest at G0/G1 phases [41]. Similarly, TPGS-coated liposomes with SiRNA-corona Bcl-2 loaded with doxorubicin (Dox) promoted a 7-fold reduction in mice tumoral mass compared to free Dox [42]. Dendrimer nanoparticles of poly(amidoamine) (PAMAM) have also been successfully used to deliver methotrexate and D-glucose (PAMAM-MTX-GLU), inhibiting MDA-MB 231 breast cancer cells [44].

In addition to their use in anti-tumorigenic therapy, nanoparticles can be applied to treat other pathologies, including atherosclerosis and Parkinson’s disease. Mannose-functionalized dendrimer nanoparticles (mDNP) have been formulated to selectively deliver LXR-L, the liver receptor ligand, to macrophages associated with atherosclerotic plaque [45]. Four weeks of mDNP-LXR-L administration in LDL-receptor knockout mice led to nearly a 10% reduction in atherosclerotic plaque, necrosis area and inflammatory response evaluated by the expression of the metalloproteinase 9 (MMP-9) gene, regulated by NF-kB, the nuclear factor kappa B. In contrast, no increases in the expression of hepatic lipogenic or plasma lipid genes were observed. Gold nanoparticles (AuNCs) containing N-isobutyryl-L-cysteine (L-NIBC) are promising in the treatment of Parkinson’s disease. An in vitro assay of the effect of 1-methyl-4-phenyliridine (MPP) on SH-SY5Y cells showed that the nanoparticles inhibited synuclein fiber aggregation and formation in the brain while also preventing the formation of Lewy bodies and the death and dysfunction of neurons, all histological characteristics observed in Parkinson’s disease [46]. These data corroborated results described in mice pre-inoculated with the neurotoxin 1-methyl-4-phenyl-1,2,3,6-tetrahydropyridine (MPTP) and treated with AuNCs, which promoted the regression of behavioral disorders determined by the open field, swimming and rotarod tests, reinforcing the potential of nanoparticles loaded with L-NIBC to treat Parkinson’s disease.

The therapeutic efficacy of several pharmaceuticals has been improved by nanoparticles displaying the high potential of drugs complexed to nanomaterials to treat neoplasms, neurodegeneration and atherosclerosis, among other physiopathological conditions (Table 1). However, despite the significant science and technology advances in obtaining nanocarriers for pharmaceuticals, the time required for optimization and regulation aiming at their commercialization can take at least thirteen years, without considering pre-clinical studies and large-scale production, as well as phase 0, I, II and III clinical trials that must precede the submission and approval of health regulatory agencies to finally reach phase IV, as defined by the American Cancer Society [51,52].

**Figure 3:**
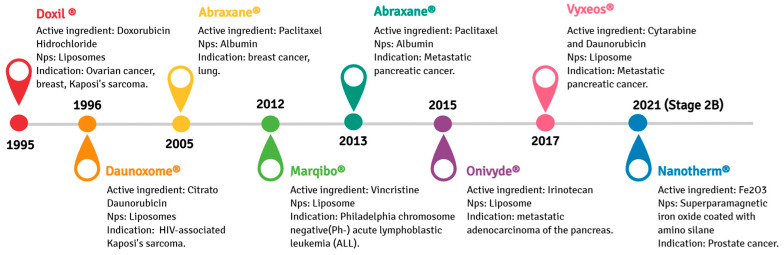
Timeline from 1995 to 2021 concerning representative anticancer pharmaceuticals loaded in nanoparticles approved by the American Food and Drug Administration for clinical purposes.

A nano-liposomal formulation of doxorubicin (DOX), Doxil^®^, was the first drug- functionalized nanoparticle formulation approved by the U.S. Food and Drug Administration (FDA) in 1995. Doxil^®^ can accumulate in solid tumors due to the EPR phenomena, effectively treating several cancers, including metastatic ovarian cancer and AIDS-related Kaposi’s sarcoma [53,54,55]. The anti-tumorigenic effect of Doxil^®^ is attributed to its DNA interleaving ability and topoisomerase II inhibition, resulting in the downregulation of DNA replication and RNA transcription. Doxil^®^ is not readily cleared from plasma by the mononuclear phagocytic system (MPS), which allows the continuous release of encapsulated DOX, improving its therapeutic performance compared to free-DOX [56,57]. Another available chemotherapy formulation is Abraxane^®^, in which paclitaxel is efficiently encapsulated in albumin nanoparticles. Abraxane^®^, approved by the FDA in 2005 and originally used to treat breast cancer, was expanded to treat advanced pancreatic carcinoma in 2013 [58]. Its formulation improves paclitaxel bioavailability, resulting in higher intra-tumoral paclitaxel concentrations facilitated by endothelial transcytosis through the albumin receptor-mediated (gp60) [59]. Other examples of nanostructured drugs developed for cancer therapy or diagnosis are depicted in Figure 3.

### 2.1. Challenges and Successful Strategies for the Therapeutic Use of Proteins and Peptides

The market for macromolecules such as proteins and peptides as pharmaceuticals has increased significantly in recent years due to their promising therapeutic benefits. Many natural peptides and proteins have been approved for clinical use, and many others are in the pre-clinical or clinical trial stages [60,61,62]. A total of 208 new drugs were approved from 2015 to 2019, 58 of which are biological compounds, including 15 peptides or peptide-containing molecules. These molecules have been applied to different clinical purposes, acting as hormones, antigens, antibodies, enzymes, vaccines and nutraceuticals for various purposes, including in the treatment of tumors, metabolic disorders and infections [63,64,65]. Although highly explored, oral delivery, the most preferred and acceptable route of administration, is limited due to poor absorption, gastrointestinal degradation and low solubility in the bloodstream and intracellular aqueous environments [66]. Thus, studies involving proteins and peptides are being performed to avoid these restrictive physicochemical characteristics [67,68,69,70].

Low-molecular-mass conventional pharmaceuticals display high cytotoxicity, as these compounds can access several cellular and intracellular compartments indiscriminately and non-specifically, triggering toxicological effects along with their health-promoting ability, as noted for conventional chemotherapeutic drugs. In contrast, high-molecular-weight proteins and peptides displaying diversified structural features, which impair their free transit in the body, present a lower probability of causing toxic effects and superior target selectivity but poor stability [5,71]. Proteins and their derivatives are very labile, degrade at room temperature and physiological conditions and are sensitive to proteases, pH variations and ionic media composition promoted by various compounds found in body fluids. Before reaching their cell or tissue targets, these molecules must also be able to overcome physiological barriers that vary according to the administration route. Through oral administration, proteins and peptides transit across the gastrointestinal tract where biochemical, mucus and cellular barriers comprise limiting factors for their stability, which justifies their poor bioavailability as low as 0.1%. In the stomach, gastric juice at extremely acidic pH values between 1.0 and 2.0 confers an optimal environment for pepsin action toward these macromolecules. Moreover, pH variations contribute to protein and peptide instability, altering their global charge and resulting in the loss of or reduction in function, structural character and solubility. Protein and peptides are exposed to pH shifts along the intestinal trait as pH 4.0–5.5 in the duodenum, pH 5.5–7.0 at the jejunum and pH 7.0–7.5 in the ileum. In the intestine, proteases such as trypsin, chymotrypsin, carboxypeptidases, elastases, aminopeptidases, endopeptidases and γ-glutamyl transpeptidases cleave peptide linkages. Additionally, to be absorbed, proteins and peptides need to reach epithelial cells by crossing a double mucus layer that lines the gastrointestinal tract, conferring a physical barrier. Mucus is a hydrogel-like substance rich in mucin and proteases, which can trap proteins and peptides, limiting their diffusion and exposing them to proteolytic activity. The portion of peptides and proteins that reach epithelial cells must still cross the cellular barrier that strictly limits molecule transport by the transcellular route to lipophilic molecules and by the paracellular route to molecules of less than 13Å, which excludes most intact macromolecules [31,52]. In the bloodstream or tissues, peptides and proteins can rapidly vanish through the classical drug clearance phenomenon or immunogenicity-driven adverse effects triggered by degradation, aggregation or post-translational modifications and antigen-based immune response [5,63,71].

Many strategies have been developed to avoid repeated administration of protein and peptide drugs and achieve their therapeutic efficacy, such as structural modifications or alterations of surrounding conditions and the use of drug carriers that may be combined with other strategies (Table 2) [72]. Their structural characteristics can be manipulated by incorporating non-usual amino acids into the peptide backbone, altering their primary amino acid sequence by altering intramolecular bonding, chemical backbone modifications and the use of PEG (Table 2). Increments in half-life, stability, receptor affinity or controlled toxicity have been observed following the incorporation of non-natural amino acids to the anti-diuretic desmopressin (DDAVP^®^), and conjugating PEG has achieved immunogenicity reductions with regard to the bovine enzyme Pegademase (Adegan^®^), used to treat severe combined immunodeficiency disease. Surrounding conditions can be modified using adjuvants such as protease inhibitors, pH modifiers, permeation enhancers, immunomodulators and hyaluronidases. Oral administration of the glucagon-like peptide (GLP-1) (Rybelsus^®^) associated with SNAC (sodium N-[8-(2-hydroxybenzoyl) amino caprylate]) neutralizes gastric juice pH, preventing peptide backbone degradation and improving transcellular drug absorption. Non-invasive alternatives for administering proteins and peptides have been developed, including the first oral glucagon-like peptide (Rybelsus^®^) and insulin-associated fumaryl diketopiperazine to produce an inhalation powder (Afrezza^®^). Moreover, improved selectivity for CD86 and CD80 receptors has been obtained by amino acid replacement in the immunosuppressor protein Belatacept (Nulojix^®^), while the use of a depot system can be applied to extend the half-life of Leuprolide (Lupron Depot^®^) [5,71].

### 2.2. Nano-Delivery Strategies for Protein and Peptide Drugs

Nano-delivery systems, particularly nano-liposomes, have been successfully applied to overcome most physiological barriers found in parenteral, oral, pulmonary, nasal, ocular or topical administrations, in combination or not with the aforementioned techniques [71,73]. The unique amphiphilic nature of liposome phospholipids allows the encapsulation of lipophilic active molecules in the lipidic bilayer, hydrophilic molecules into the aqueous core and amphiphilic compounds in the interface. Moreover, nano-capsules can be functionalized with surface molecules aiming at targeted delivery, immune system evasion, stability or other specific purposes. Additional advantages offered by this drug delivery system include biodegradability, biocompatibility and low immunogenicity, with insignificant toxicity, configuring a promising nanocarrier for macromolecules [74].

As demonstrated for other natural compounds, proteins and peptides can be encapsulated within colloidal particles absorbed by cells, subsequently releasing the active principle and reaching systemic circulation [67,70,75]. Additionally, these macromolecules can be conjugated with ligands on the surface of nanoparticles to facilitate intestinal absorption, targeting intestinal receptors or transporters [76,77]. Some protein-loaded liposomes have been approved for clinical use, with many others in the pre-clinical stage, delineating a hopeful future for the efficient and specific targeting of protein and peptide drugs [62].

The first use of proteins in association with a nano-liposomal system approved by the FDA in 1994 was in the vaccine against hepatitis A (Epaxal^®^), where virus-derived proteins are incorporated onto liposome surfaces, targeting the nanocarrier to immune cells. The same strategy was applied to flu vaccines in the 1990s. Curosurf^®^ is a suspension that contains a mixture of phospholipids and two surfactant proteins, SP-B and SP-C, marketed in particle sizes from 30 µm to 50 nm to treat neonatal respiratory distress syndrome, preventing alveolar collapse [62]. Polymeric nanoparticles are also employed to deliver the growth-colony stimulating factor, Interferon-α2a and L-asparaginase to modulate the immune system, treat infections and against cancer (Table 2).

Many other promising possibilities, still under investigation, are now being reported. Lactoferrin (Lf), for example, has been complexed to doxorubicin (DOX) to lead this drug to hepatocellular carcinoma (HCH) cells, where Lf specifically interacts with the asialoglycoprotein receptor (ASGPR) leading to an antitumoral response [77]. Mice treated with pegylated liposome (Lf-PLS) loaded with DOX displayed superior delayed growth of HepG2 cells compared to DOX-loaded PLS and free DOX, improving the absorption by target cells and resulting in an inhibitory effect on ASGPR-positive HCC cell lines. The results indicate that Lf-PLS loaded with DOX may be a potential drug-targeting delivery system for HCC treatment. Moreover, Lf per se is a bioactive protein encapsulated in nano-emulsions [78] and liposomes [79,80], among others. Simulated digestion of encapsulated lactoferrin (LF) has demonstrated that this compound is protected against gastric juice degradation, with no apparent damage to the liposome bilayer structure, even under low pH and pepsin proteolysis conditions [79,80]. Based on this, this formulation seems promising for the oral administration of proteins and peptides, as lactoferrin and other proteins mentioned below exhibit immunomodulatory properties and lead to anti-proliferative effects by inducing apoptosis in cancer cells [81,82,83,84].

Bromelain nanoencapsulation, for example, improved the anti-tumorigenic effect of this compound by prolonging its release in various tumor cell lines, including U251; MCF-7; OVCAR-03; NCI-ADR/RES; NCI-H460; PC-3; HT-29; K562; and HaCaT and healthy human cells and keratinocyte migration and proliferation in the scratch assay. Cell migration was inhibited by 90% after 24 h [85].

Promising anticancer effects of nano-encapsulated proteins have also been observed for taro lectin, tarin, coated by pegylated nano-liposomes. The pharmacological properties of tarin seem to be preserved, whereas bioavailability and therapeutic effectiveness were improved. Nano-liposomal tarin, a lectin isolated by our group from a *Colocasia esculenta* extract, inhibited human cancer cell lines such as glioblastoma (U-87 MG) and adenocarcinoma (MDA-MB-231) with a CI_50_ of 39.36 μg/mL and 71.38 μg/mL, respectively, comparable to the conventional chemotherapeutics cisplatin and temozolomide. Furthermore, no toxicity was observed against healthy mouse L929 fibroblasts and bone marrow cells. Although no inhibition was detected for free tarin up to 50 μg/mL after 24 h of exposure, no activity was observed for empty liposomes, and thus, antitumoral ability can be attributed to tarin only [86,87,88,89]. Moreover, taro antitumoral activity against MDA-MB-231 and other cancer cell lines has been demonstrated and attributed to tarin in previous studies [90,91]. Similarly, the lectin from *Lepidium sativum* encapsulated in chitosan exhibited significant cytotoxic effects against hepatocellular carcinoma cells (HepG2) in a dose-dependent manner, reaching up to 66% inhibition [92].

Nanoencapsulation has also proven a suitable technique for developing anticancer peptides. Bioactive peptides from rice husks encapsulated in chitosan, at 0.1%, 0.2% and 0.3% (*w*/*v*) protein hydrolysates, for example, gradually suppressed the cellular viability of several human tumorigenic cell lines, such as A549, MDA-MB-231 and MCF7, while VERO cells, a non-carcinogenic line, were not affected by the protein hydrolysates, indicating that peptides within the peptide pool were released [93].

## 3. Liposomal Formulation Performance: Characteristics, Functionalization and Internalization

An effective liposomal formulation can be achieved by choosing the suitable lipid composition, proper functionalization and a smart targeting strategy. Phospholipid selection, considering the head group and chain length and number of liposome components, are crucial in determining liposome safety, stability and efficiency [94]. Besides their chemical composition, liposome efficiency depends on their stiffness, surface charge and lipid organization, as surface modifications interfere with liposome stability and can be handled to expand its use [95,96].

The main liposome components are glycerophospholipids, amphiphilic lipids composed of a glycerol molecule linked to a phosphate group and two fatty acid chains that can be either saturated or unsaturated. Phosphatidylcholine and phosphatidylethanolamine, abundant in plants and animals, are the most employed to form liposomes [97,98]. Liposomes can acquire positive, negative or neutral charges depending on the phospholipid head and chain, which confer their overall characteristics and functionalities [99,100]. Stability can be conferred to liposomes formed by phospholipids with longer tails and low degrees of unsaturation and ether linkages. Phospholipids with longer saturated hydrocarbon chains display a remarkable ability to interact with each other and form tightly ordered bilayer structures. On the other hand, shorter unsaturated hydrocarbon chains form liposomes with fluid and disordered bilayers [94,101].

Synthetic phospholipids can be formed by the modification of the head groups, aliphatic chains and alcohols of natural phospholipids, creating an enormous variety of synthetic phospholipids, such as 1,2-distearoyl-sn-glycero-3-phosphocholine (DSPC), 1,2-dipalmitoyl-sn-glycero-3-phosphocholine (DPPC), 1,2-dioleoyl-glycero- 3-phosphocholine (DOPC), 1,2-distearoyl-sn-glycero-3-phosphoglycerol (DSPG), 1,2-dipalmitoyl-sn-glycero-3-phosphoglycerol (DPPG), 1,2-dioleoyl-sn-glycerol -3-phosphoethanolamine, (DOPE) and 1,2-distearoyl-sn-glycero-3-phosphoethanolamine (DSPE), which have proven to be more stable [102].

Liposomes are formed by the hydrophilic interactions between polar head groups, the van der Waals forces between hydrocarbon chains, holding the long hydrocarbon tails together, and hydrogen linkages with H_2_O. In turn, H_2_O repels the hydrophobic chains, and the liposomes self-assemble spontaneously into a closed bilayer [102,103].

**Figure 4:**
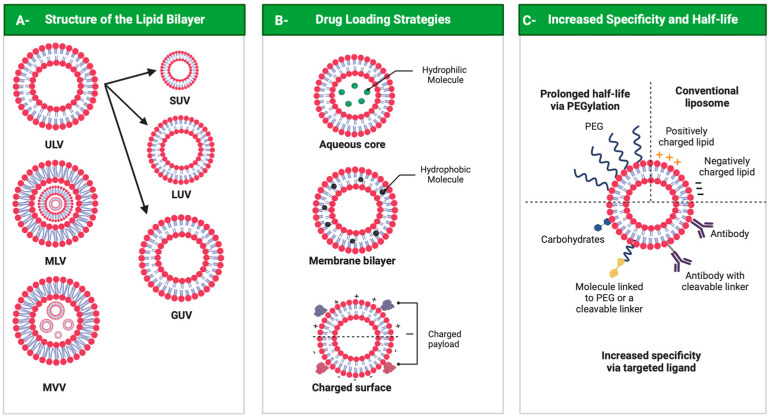
Liposomes. Panel A: Structure of liposomes. ULV—unilamellar vesicle (small ULV (SUV), large ULV (LUV) and giant ULV (GUV)); MLV—multilamellar vesicle; MVV—multivesicular vesicle. Panel B: Drug loading strategies. Panel C: Liposome surface and functionalization. All figures were drawn with BioRender accessed on 1 December 2022.

Liposomes can be classified as neutral, anionic and cationic according to lipid bilayer components. They can assume different 3D structures, as unilamellar vesicles (ULVs), multilamellar vesicles (MLVs) or multivesicular vesicles (MVVs). ULVs contain a single-lipid-bilayer membrane and can vary in size, with small unilamellar vesicles (SUVs) measuring between 30 and 100 nm, large unilamellar vesicles (LUVs) from 100 to 300 nm and giant unilamellar vesicles (GUVs) from 1 to 100 μm. In multilamellar vesicles, layers are concentric, while in MVVs, several smaller vesicles encase the interior of another vesicle (Figure 4, panel A) [104,105]. Another liposome formed by two bilayer membranes, the double-layer vesicle, comprises a framework that can improve liposome stability, which delays and sustains the release of their load [106].

In addition to phospholipids, its main components, cholesterol, glycol-derivatives including propylene glycol and polyethylene glycol (PEG) and even polymers such as chitosan, can increase liposome stability. These components can promote pronounced effects on healthy tissues and cells and activate or suppress immune responses [107,108]. Cholesterol incorporation into liposome bilayers can influence their fluidity, reducing their permeability and increasing in vitro and in vivo stabilities. Cholesterol, a hydrophobic molecule, induces a dense phospholipid packing and inhibits interactions among lipid chains interspersed between them, promoting liposome membrane stabilization [109,110,111]. The cholesterol molecule accommodates its hydroxyl group close to the hydrophilic region of phospholipids, and its aromatic ring lays parallel to the fatty acid chains in the lipid bilayer [112]. In the absence of cholesterol, liposomes can interact with proteins such as albumin, transferrin, macroglobulin and high-density lipoproteins (HDL), destabilizing the liposomal membrane structure and, consequently, decreasing drug delivery system performance [113,114,115].

Polymers such as chitosan are also used for liposomal surface modification, leading to a protective shell on the liposome surface, mainly for oral drug delivery [108,116]. Glycols such as propylene glycol incorporated into phospholipid vesicles with polyethylene glycol (PEG) have been advocated as flexible lipid vesicles to improve drug delivery systems targeting the skin [117,118]. Different PEGs on liposome surfaces can prolong their half-lives in the bloodstream, from a few minutes considering conventional liposomes to several hours for stealth liposomes, also called PEGylated liposomes [112]. One of the main disadvantages of conventional liposomes is their rapid elimination from the bloodstream, and they arrive in organs and tissues of the reticuloendothelial system, such as the liver and spleen [119]. The increases in liposome-circulating lifetimes promoted by PEG depend on the amount of grafted PEG and the molecular weight of the polymer. PEG longer chains typically increase the bloodstream residence time, reported as higher for PEGylated liposomes containing PEG 1900 and PEG 5000 compared to PEG 750 and PEG 120 [120,121]. Their molecular weight determines the conformation of PEG polymers on the surface of liposomes and PEG surface density in mushroom (low concentration) or brush (high concentration) regimes [122]. PEG concentrations from 5% to 10% (molar ratio) result in improvements in the degree of liposome stealth, and higher PEG concentrations (brush regimen) input to liposomes lead to high resistance to phagocytosis and poor activation of the human complement system [123].

PEG-enhanced liposome surfaces are associated with a cloaking effect, mimicking water-like structures, thus providing a steric barrier that prevents protein adsorption to liposome surfaces and their recognition by the mononuclear macrophage phagocytic system that would otherwise lead to rapid liposome clearance [94,102]. On the other hand, repeated venous administrations of PEGylated liposomes in animals at certain intervals induce immune responses, resulting in the loss of long-circulating characteristics and accelerating the blood clearance (ABC) phenomenon [124,125]. It has been suggested that anti-PEG IgM, produced by the spleen in response to a first dose, selectively binds to PEG chains in a second dose administered several days later and subsequently activates the complement system, one of the main opsonins, increasing the liver uptake of the following doses [125,126]. The occurrence and magnitude of the ABC phenomenon are influenced by the dose and physicochemical properties of PEGylated liposomes and the time interval between liposome administration and the encapsulated drug [127]. Many approaches have been tested to minimize the immunogenicity of the PEG moiety following repeated administrations. PEG lipids presenting a shorter alkyl chain can dissociate more quickly from the lipid bilayer, such as mPEG-DSPE and mPEG-CH, attenuating the ABC phenomenon [128,129].

### 3.1. Liposome Functionalization

Since their discovery, liposomes have been produced with different characteristics based on their composition and functionalization. The first generation of liposomes designed for therapeutic use are termed conventional liposomes (Figure 4, panel C) [120,130,131]. These liposomes were neutral-, cationic- or anionic-charged phospholipids, usually combined with cholesterol, to promote liposomal bilayer stabilization [102,132]. However, their short life in the bloodstream due to rapid capture by the reticuloendothelial system is very inconvenient [120]. Binding of opsonins that recognize liposomes as foreign particles is the first signal for liposome elimination from plasma by phagocytosis in the mononuclear phagocytic system [133].

The second generation of liposomes created stealthy, long-lasting and/or PEGylated liposomes aiming to improve their performance, as mentioned previously. This strategy mainly involves coating the surface of the liposomal membrane with biocompatible hydrophilic polymer conjugates such as PEG, chitosan and others, increasing the repulsive forces between liposomes and other serum components (Figure 4, panel C) [134]. This strategy reduces immunogenicity and macrophage uptake, increasing their half-life in the bloodstream and reducing the toxicity of the encapsulated compound [94,102]. Methods for anchoring PEG to the liposome membrane involve physical adsorption of the polymer onto liposome surfaces, incorporation of the PEG-lipid conjugate during liposome preparation or the covalent attachment of reactive groups on the surface of pre-formed liposomes [120]. However, a significant restriction of stealth liposomes is their large body biodistribution, not selectively delivered to specific target cells [135]. Due to this limitation, ligand-targeted liposomes were designed to orientate compound delivery to target tissues, improving therapy selectivity [120]. In addition to PEG, liposomes can be functionalized by attaching glycoproteins, polysaccharides or ligands for specific receptors, such as antibodies, small molecules or peptides [133,135]. The ligand can target specific receptors overexpressed on the surface of unhealthy cells, binding to them and resulting in minimal off-target effects [136,137].

The design of liposomes functionalized by antibodies called immunoliposomes, and liposomes responsive to stimuli, is also being considered (Figure 4, panel C) [138]. Immunoliposomes functionalized by the chemical coupling of antibodies or their fragments result in high-specificity target antigens. [139]. In stimulus-sensitive liposomes, drug release occurs through physical-chemical changes or biochemical stimuli, such as pH, temperature, redox potential, enzyme and electrolyte concentrations, ultrasound and electric or magnetic fields [140,141]. The most common stimulus-responsive liposomes are pH- and/or temperature-sensitive liposomes [37,142].

Multifunctional liposomes display modified surfaces aiming at multiple functions resulting in liposomes with a wide range of functionalities [133]. Theranostic liposomes are nanoparticles coating the bioimaging compound and the therapeutic agent applied for diagnosis and treatment [132,143]. Dual-targeting liposomes are functionalized with two different ligands that can also be successfully designed [133].

### 3.2. Liposome Internalization and Delivery Mechanism

**Figure 5:**
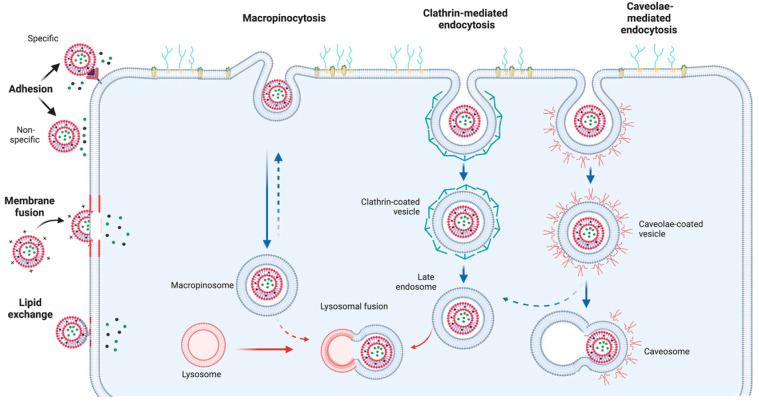
Liposome internalization and delivery mechanisms. The figure was created with BioRender accessed on 1 December 2022.

The poor knowledge of the mechanisms involved in cellular liposome internalization has impaired advanced drug delivery by these nano-devices to treat neoplastic diseases, boost innate immunity and even, in gene silencing, hinder or block the production of harmful proteins, including transcriptional factors. The internalization of this nanosized particle in the target cell occurs through endocytosis, where the cell membrane surrounds the nano-device in some sites and engulfs it into a vesicle that is detached from the cell membrane, penetrates the cytosol and continues its route to the intracellular target organelle after overcoming endosomal entrapment [144,145] (Figure 5). Conventional classification of endocytic pathways is divided into phagocytosis, related to the internalization of macromolecular structures through the invagination of the cell membrane, or pinocytosis, regarding the internalization of nanosized particles. Pinocytosis is classified according to the coating proteins on the endocytic particle in a clathrin-dependent or clathrin-independent way, which can be mediated by caveolae. The clathrin-mediated endocytic pathway can also involve the dynamin, a large GTPase that assembles into polymers on the budding membranes and mediates fission during the clathrin-dependent process [144,146]. Using a set of pharmacological inhibitors such as concanavalin A, chlorpromazine, dynasore, genistein, filipin, MBCD, nystatin, EIPA and cytochalasin D has shed some light onto nanocarrier transport mechanisms. The internalization of liposomes and their cargo delivery are inhibited by MBCD and nystatin, where both affect cholesterol in plasma membranes, and EIPA, a macropinocytosis inhibitor that impairs Na+/H+ exchanges and lowers the cell cytoplasm pH. Chlorpromazine has also demonstrated an effect on liposome internalization, indicating that the clathrin-mediated (dynamin-dependent) is involved in nano-liposomes endocytosis. However, this endocytic mechanism seems not to involve the G-protein-coupled receptors in plasma cell membranes, which should prevent the assembly of clathrin-coated pits and impair their movement through the cell membrane, a mechanism deduced because another clathrin-mediated endocytosis inhibitor, concanavalin A, has not displayed a significant effect on liposome internalization. Macropinocytosis-dynamin-independent and cell-membrane-cholesterol-dependent clathrin- and caveolae-independent processes are shown to play a role in liposome internalization (Figure 5) [147].

Adhesion comprises an interaction mechanism between liposomes and the cell surface, without internalization that can occur in two ways, namely by specific adhesion, where specific proteins in the liposomal membrane bind to the corresponding receptors on the cell surface, and nonspecific adhesion, which occurs when attractive forces exceed repulsive forces. Adhered liposomes can lead to high local liposomal content near the cell surface, destabilizing liposomes and promoting the leak of liposomal loads into target cells (Figure 5) [148,149].

An additional mechanism consists in the fusion between the plasma membrane and the liposome membrane, where liposomal lipids rapidly fuse with the plasma membrane by lateral diffusion, and the lipids and liposome contents are delivered directly into the cell (Figure 5) [150,151].

Lipid exchange can be involved in exchanging similar lipid molecules between liposomes and target cell membranes without participating in the liposomal cargo [148]. Indeed, this long-term interaction between cell membrane phospholipids is recognized by lipid transfer proteins that facilitate lipid exchanges [152]. The exchange process results in low liposome stability and the liposome load entering the target cell. During the lipid transfer process, lipids in the liposome are preferentially introduced into the outer leaflet of the plasma membrane [153]. Plasma membrane cholesterol can reduce lipid exchange, as it improves the mechanical stability of the bilayer by reducing fluidity and permeability (Figure 5) [151].

### 3.3. Liposomal Formulations in Antitumorigenic Drugs and Vaccines: Marketed or Phase III Clinical Trial Products

Liposomes successfully entered the market in 1995 with the development of the PEGylated Doxil^®^ liposomal formulation, as mentioned previously. Since then, no setbacks to these delivery systems have been noted, which have been explored concerning several physiopathological conditions, from cancer treatments to vaccine formulations.

Doxil^®^, the formulation containing DOX hydrochloride as the active agent, is the first FDA-approved nano-drug delivery system regarding PEGylated liposome technology [154]. Doxil^®^ liposomes are formed by high-temperature phase transition (Tm) components, including hydrogenated soy phosphatidylcholine (HSPC), cholesterol and N-(carbonyl-methoxy-polyethylene glycol 2000)-1,2-distearoyl-sn-glycero-3-phosphoethanolamine sodium salt (MPEG-DSPE) at a molar ratio of 56:38:5 [155]. Better drug retention has been obtained by employing optimal cholesterol: HSPC ratio, which forms a non-flexible bilayer at 37 °C and below. DSPE is incorporated into the liposome bilayer to provide a reactive functional group for the hydrophilic chains of PEG 2000, covalently bound to the DSPE head that elongates into the inner and outer water phases. The overall lipid content of Doxil^®^, in the hydrophilic core of liposomes, is approximately 16 mg/mL, and DOX at 2 mg/mL is in the hydrophilic core of liposomes complexed to sulfate salt [156]. Over 90% of DOX is encapsulated in tiny, unilamellar, 80–100 nm liposomes [154]. A high and stable drug/lipid ratio is obtained due to the ammonium sulfate transmembrane gradient between [(NH_4_)_2_SO_4_] liposome >> [(NH_4_)_2_SO_4_] medium, the driving force for the efficient and stable loading of amphipathic agents into liposomes [157]. The accumulation of 15,000 DOX molecules within the hydrophilic core of each liposome adds to >90% of DOX at a crystalline precipitate, protected from physicochemical constraints, contributing to the stability of drug trapping [158,159]. Doxil^®^ formulations display significant drug retention and low drug efflux into the circulation, allowing acceptable drug delivery rates to target tissues [160].

Another liposomal doxorubicin nanoencapsulation was formulated to confer free doxorubicin more tolerable and improved effectiveness, giving rise to MM-302 and ThermoDox^®^. The MM-302 formulation is a HER2-targeted liposomal doxorubicin-antibody conjugate composed of DSPE and PEG that targets specific cells that overexpress HER2 and increase doxorubicin delivery to tumor cells, limiting exposure of healthy cells such as cardiomyocytes. The MM-302 formulation plus trastuzumab at 30 mg/m^2^  and 14 mg/kg IV Q3W were evaluated in a phase II clinical trial in patients with localized advanced and/or metastatic HER2-positive breast cancer [161]. ThermoDox^®^ is the first heat-activated liposomal drug carrier formulation to be used to treat solid tumors under clinical trials [162]. This unique formulation was designed for long-circulating time, and its smooth thermo-sensibility has been applied in a clinical study in combination with radiofrequency ablation to remove the tumor core. This therapy was indicated for primary liver cancer, hepatocellular carcinoma and recurrent chest wall breast cancer. These liposomes are composed of DPPC, myristoyl-stearoyl-phosphatidylcholine and 1,2-distearoyl-sn-glycero-3-phosphoethanolamine-N-[amino(polyethyleneglycol)-2000] (DSPE-PEG-2000), and the three lipids are combined to achieve a sharp thermal transition and rapid membrane permeability onset [163]. This specific combination led to a patented heat-activated DOX liposome named thermoDox^®^ [164,165]. DPPC displays a transition temperature of 41.5 °C and undergoes a phase shift at 42 °C, reached by local hyperthermia during clinical treatment. Adding myristoyl-stearoyl-phosphatidylcholine to the composition accelerates the drug release due to a slight reduction in DPPC transition temperature, while DSPE-PEG-2000 increases liposome circulation times, as expected. The presence of PEG-lipid also helps achieve faster lysolipid-induced permeability. Due to initially promising results, the phase III clinical trial began in 2009, termed the “HEAT” trial (NCToo617981). Clinical trials were employed to determine whether the use of ThermoDox as an adjunct to radiofrequency ablation would lead to additional benefits compared to radiofrequency ablation alone. HEAT, however, did not meet its goal of progression-free survival. However, analyses concerning patient subgroups revealed a therapeutic benefit for ThermoDox in patients who received prolonged radiofrequency ablation treatments (with a minimum dwell time of 45 min). Based on these promising findings, a new phase III clinical trial (the OPTIMA trial) began in 2014. OPTIMA differed from the HEAT trial in presenting a standardized warming protocol, which included a minimal radiofrequency ablation dwell time of 45 min (NCT02112656). However, OPTIMA demonstrated that the addition of ThermoDox to radiofrequency ablation does not provide a measurable survival benefit over radiofrequency ablation alone [166].

DaunoXome^®^ is a liposomal daunorubicin formulation developed in 1996 for treating HIV-associated Kaposi’s sarcoma. Due to the small size of nano-liposomes, between 45 and 80 nm, the uptake of DaunoXome by the reticule-endothelial system is diminished, leading to extensive drug circulation. DaunoXome has a half-life between 4 and 5.6 h, much longer than free daunorubicin, at about 0.77 h [167]. Each single-dose vial contains approximately 50 mg of daunorubicin (DNR) in liposomes composed of 168 mg of cholesterol and 704 mg of distearoyl-phosphatidylcholine [168]. Onivyde™, a rinotecan liposome injection coupled to leucovorin and fluorouracil, is indicated for treating patients affected by metastatic pancreatic adenocarcinoma who experience disease progression following gemcitabine therapy. Onivyde™ is formulated in a liposomal dispersion with a water-soluble semi-synthetic irinotecan hydrochloride trihydrate, a topoisomerase inhibitor drug. Onivyde™ liposomes are unilamellar 100 nm lipid layer vesicles encapsulating irinotecan in a gelled or precipitated state, such as the sucrose octasulfate-salt, using an aqueous space titration/ion exchange method through intra-liposomal drug stabilization technology [169]. Highly charged polymeric or non-polymeric anions and intra-liposomal capture agents, such as polyphosphate or sucrose octasulfate-salt, have been combined to a poly-alkylamine gradient. Irinotecan is encapsulated in a nano-liposome composed of distearoyl-phosphatidylcholine, cholesterol and methoxy-terminated polyethylene glycol distearoyl-phosphatidylethanolamine (MPEG-2000-DSPE) at a 3:2:0.015 ratio. The drug encapsulation efficiency achieved 90%, reaching a ratio of 800 g irinotecan per mole of phospholipid within liposomes. The half-life of the drug delivered by this system was 56.8 h following administration [170].

In addition to drug delivery, cationic liposomes can be used as transfection vectors for gene therapy [138]. The cationic liposome coat can protect loaded nucleic acids (DNA or RNA) against nuclease degradation during storage and in the bloodstream [120]. An example of nucleic acid encapsulation in lipid nanoparticles is the Tozinameran vaccine (trade name: Comirnaty^®^), developed by Pfizer/BioNTech that received Emergency Use Authorization by the United States Food and Drug Administration (FDA) in December 2020, aiming at the prevention of coronavirus disease 2019, COVID-19. In its pre-fusion stabilized form, the mRNA encoding the SARS-CoV-2 spike protein was used to induce neutralizing antibodies [171]. mRNA coated by lipid nanoparticles is successful when administered intramuscularly in humans at 30 μg in a series of two doses (10 μg/0.1 mL) three weeks apart [172]. Elasomeran (trade name, Spikevax), developed by Moderna, received the US emergency authorization use at the same time as Tozinameran in 2020 [173]. The two lipid nanoparticle vaccines share several similarities in their formulation and behave similarly in vivo to that expected for nanoparticles. It is important to note that the lipid nanoparticles in both formulations are composed of ionizable lipids, phospholipids, cholesterol and PEG-lipids. The two ionizable lipids, ALC-0315 and SM-102, contain a tertiary amine group with pKa between 6.0 and 6.7 that alter their charges from neutral to cationic according to bloodstream or endosome pH. The two PEG-lipids, ALC-0159 and PEG200-DMG, contain 14-carbon-long di-alkyl chains, which aid in their rapid dissociation from the surface of lipid nanoparticles once inside the human body [174,175,176]. Since ideal drug carriers are quickly eliminated from the body once their purpose is fulfilled, several lipid nanoparticles have incorporated biodegradable designs into ionizable lipids to facilitate their elimination [177,178,179]. Intramuscular injections of mRNA-lipid nanoparticles composed of SM-102 result in faster clearance alongside better injection site tolerability [176]. The two ester bonds in ALC-0315 are also hydrolyzed in vivo [180].

Comparing the lipid compositions of Tozinameran and Elasomeran, the former contains 3.23 mg of ionizable lipids, ALC-0315, 0.7 mg of phospholipids, 1.4 mg of cholesterol and 0.4 mg of PEG-lipid (ALC-0159). Elasomeran comprises 1.075 mg of ionizable lipids, the SM-102, 0.275 mg of phospholipids, 0.47 mg of cholesterol and 0.115 mg of PEG-lipid, the PEG_200_-DMG. Although the main difference between the two formulations is vaccine storage conditions, as Elasomeran requires freezing temperatures between −50 °C and −15 °C, and Tozinameran requires deep freezing temperatures between −90 °C and −60 °C, it is believed that mRNAs, not lipid nanoparticles, are the main factors for the short stability of these vaccines, so ultra-freezing temperatures are required to delay the degradation of the mRNA load [181].

### 3.4. Limitations concerning the Application of Nano-Liposomes in Drug Delivery

Although liposome-based delivery systems were discovered in 1965, the first FDA-approved drug product dates from 1995, comprising the liposomal doxorubicin (Doxil), and even today, with a vast amount of accumulated data and knowledge concerning nano-liposomal delivery systems, the number of approved drugs does not increase in the same proportion. Some of the limitations that impair the success of new nano-liposomal formulations during clinical trials comprise stability issues, high production costs, activation of deleterious immune responses and biodistribution problems [182,183].

Incorporating PEG onto liposome surfaces has proved to be an efficient physical barrier to prevent degradation, liposome aggregation, clearance and drug leakage and size maintenance by impairing enzymes and antibody access. Consequently, liposome half-lives in the bloodstream are improved. On the other hand, PEG limitations include immunogenicity hypersensitivity and non-degradability. Complete evasion from macrophages and other immune cell recognition are not achieved, leading to anti-PEG antibody releases that affect the long-term circulation of PEG-coated liposomes through clearance acceleration. Moreover, repeated administration of pegylated liposomes can lead to in vivo accumulation due to its systemic non-degradability. Non-degradable and degradable alternatives have been extensively studied, including alginate, hyaluronic acid, Poly(vinyl pyrrolidone), Polyglycerol and others [73].

Poor encapsulation of hydrophilic drugs and storage instability associated with drug leakage is another obstacle, which can be overcome by the use of other lipid-based nanoparticles such as niosomes, transferesomes, solid-lipid nanoparticles and nanostructured lipid carriers [184].

Besides using non-eco-friendly solvents, the methodologies generally employed for nano-liposome production require specialized personnel and strict control of the multiple process steps to guarantee reproducibility and the production of correct liposomal features. More lean and robust processes such as preparations based on microfluidics have demonstrated successful and promising performance, although other limitations are noted [185].

The behavior and functionality of nano-liposomes across animal and human species can differ, which is translated into clinical trial failures, which can also be influenced by patient heterogeneity. Therefore, understanding the relationship between nano-liposomes and human physiology and pathologies is crucial to achieve optimized and successful performance. Currently, the engineering of precision nano-delivery systems through functionalization by combining target-specific molecules and bio-responsive moieties represents the best choice to improve efficacy and patient outcomes [186].

## 4. Conclusions

Nano-devices, in particular, nano-liposomes, have been increasingly considered great allies in immunotherapy and regenerative medicine. The uptake of liposome-carrying pharmaceuticals by cells occurs mainly by two mechanisms, endocytosis and membrane fusion. If liposome uptake takes place through a membrane fusion process, liposomes can release their loads into the cytoplasm of target cells, whereas endocytosis must be followed by liposome escape from the endosomal multivesicular pathway in order to release their loads into the cytosol of target cells, which will eventually be re-directed into a sub-cellular structure. Alternatively, liposomes can release their loads through a membrane fusion process. Nano-liposomes are loaded with drugs or biomolecules, which, when delivered may regulate cell functions or trigger immune responses. Liposomes are non-toxic, as their structures are formed by physiological and degradable lipids, phospholipids and cholesterol, commonly found in human cell membranes, but can be PEGylated to extend liposome half-lives in the bloodstream. Liposome surfaces should be tailored to decrease their immunogenicity, but the possibility of reaching specific tissues or cells can be achieved if their surfaces carry recognizers for the epitopes present in target cells, particularly tumorigenic ones. Nano-liposomes can deliver drugs and/or active compounds irrespective of compound chemical structure or solubility, and even in high concentrations without side effects to healthy cells, which can be reduced or minimized by the coated protection of the double lipid bilayer. Currently, most marketed nano-liposomes used in cancer therapeutics are loaded with conventional anti-neoplastic drugs. Although anti-neoplastic nano-liposomes can reduce side effects in healthy cells, the absence of toxicity may be achieved if conventional pharmaceuticals are altered by novel antitumoral compounds, natural and less toxic but more effective than conventional antitumoral or immuno-stimulator compounds. The development of the newest generation of liposomes loaded with bio-molecules such as protein and peptides may be the next generation of anticancer and immune modulator pharmaceuticals, devoid of toxicity but highly effective.

## Figures and Tables

**Table 1:** Pre-clinical studies on nano-delivery systems demonstrated in cell cultures and animal models for the loading of therapeutics aiming at the treatment of several health disorders.

Nano-Delivery System	Therapeutic Agent	Loading Mechanism	Pathology	Biological Assay	Pharmacological Response	Ref.
Nanostructured lipid carriers (NLCs)	Tocotrienol/ Simvastatin	Core co-encapsulation	Mammary adenocarcinoma	In vitro (+SA lineage)	Improved anti-proliferative TRF and SIM effect upon encapsulation	[37]
Solid lipid nanoparticles (SLN)	Linalool	Encapsulation	Hepatocarcinoma Lung adenocarcinoma	In vitro (HepG2 and A549 cell lineages)	Improved cytotoxic effect on human lung- and liver-derived tumor cells (A549 and HepG2) at > 1.0 mM in a dose/time-dependent manner	[38]
Lipid nano-capsules	Simvastatin	Encapsulation	Breast carcinoma	In vitro (MCF-7 lineage)	Increased cytotoxicity at IC_50_ = 1.4 ± 0.02 mg/mL	[39]
Folic acid-chitosan	Vincristine	Encapsulation	Non-small-cell lung cancer (NSCLC)	In vitro (NCI-H460 lineage)	Anticancer activity at a 4:25 formulation against non-small-cell lung cancer (NCI-H460).	[40]
Liposomes	(III) complexes	Encapsulation	Several types of cancer	In vitro (HepG2; HTC-116; HeLa; A549; BEL-7402; SGC-7901; Eca-109; B-16 and human liver cell L02) In vivo (mice)	Ir-1-Lipo and Ir-2-Lipo induced apoptosis at 55.6% and 69.3% levels. Improved anticancer activity against A549 cells; Ir-2-Lipo effectively inhibited tumor growth in a murine model	[41]
PGS-coated cationic liposomes with Bcl-2 siRNA-corona	Doxorubicin (Dox)	Electrostatic adsorption	Hepatocellular carcinoma	In vitro (Bel7402 sensitive cells and Bel7402/5-FU MDR cells) In vivo (mice)	7-fold improved anticancer effect by apoptosis induction and tumor growth inhibition compared to free Dox	[42]
Poly lactic-co-glycolic acid (PLGA) nanofibers (NFs)	Metformin	Encapsulation	Lung adenocarcinoma	In vitro (A549 cell lineage)	Significant cytotoxicity against A549 cells by apoptosis induction	[43]
Polyamidoamine (PAMAM) dendrimers	Methotrexate (MTX) and D-glucose (GLU)	Encapsulation	Breast cancer	In vitro (MDA MB-231 lineage	OS-PAMAM-MTX-GLU displaying higher anticancer potential compared to free MTX after a 4 h exposure without significantly affecting healthy human HaCat cells	[44]
Polyamidoamine (PAMAM) dendrimers	Liver-x-receptor (LXR)	Specific receptor binding	Atherosclerosis	In vitro (mouse peritoneal macrophages) In vivo (mice)	mDNP-LXR-L-mediated delivery reduced in the expression of metalloproteinase 9 (MMP-9); followed by plaque size reduction and decreased necrosis	[45]
Gold nanoclusters (AuNCs)	N-isobutyryl-L-cysteine (L-NIBC)	Au-S bond	Parkinson’s disease	In vitro (PC12 and SH-SY5Y lineages) In vivo (mice)	AuNCs exhibited superior neuroprotective effects in 1-metil-4-phenilyridine (MPP+) lesioned cell and 1-methyl-4-phenylpyridine (MPTP) induced mouse PD models	[46]

PD—Parkinson’s disease.

**Table 2:** Representative non-nano- and nano-delivery systems for protein/peptide drugs approved by the American Food and Drug Administration or European Medicines Agency to treat health disorders.

Therapeutic Indication	Marketed Protein and Peptide Drugs	Active Principle	Delivery Strategy	Administration Route
Cancer	1- Lazertinib (leclaza^®^) 2- Pegaspargase (Oncaspar^®^) 3- Mepact^®^	1- EGFR-tyrosine kinase inhibitor 2- L-asparaginase 3- Muramyl tripeptide phosphatidyl ethanolamine	1- Amino acid modification 2- Polymeric nanoparticle (PEG) 3- Liposome encapsulation	1- Oral 2- IM/IV 3- IV
Diabetes	1- Insulin degludec Tresiba^®^ 2- Lixisenatide (Adlyxin^®^)	1- Insulin 2- Glucagon-like peptide-1 receptor agonist	1- Amino acid modification 2- Amino acid modification and amidation	1- SC 2- SC
Immune modulation	1- Belatacept (Nulojix^®^) 2- Pegfilgrastim (Neulasta^®^) 3- Sandimmune Neoral^®^	1- CTLA4 antibody 2- G-CSF 3- Cyclosporine A	1- Amino acid substitution 2- Polymeric nanoparticle (PEG) 3- Lipid-based formulation	1- IV 2- on-body injection 3- oral
Infection	1- Bezlotoxumab (Zinplava^®^) 2- Ibalizumab-uiyk (Trogarzo^®^) 3- Peginterferon-α2a Pegasys^®^	1- Monoclonal antibody against *Clostridium difficile* toxins A and B 2- Monoclonal antibody CD4-directed 3- Interferon-α2a	1- Natural 2- Natural 3- Polymeric nanoparticle (PEG)	1- IV 2- IV 3- IV

Approved by the EMA—European Medicines Agency; CTLA4—cytotoxic T-lymphocyte antigen 4; EGFR—epidermal growth factor receptor; G-CSF—growth-colony stimulating factor; IM—intramuscular; IV—intravenous; SC—subcutaneous.

## Data Availability

Data supporting reported results can be found in the manuscript.
